# Vanishing solitary osteochondroma of humerus following trauma: A case report

**DOI:** 10.1016/j.tcr.2023.100874

**Published:** 2023-06-08

**Authors:** Isam Sami Moghamis, Ahmed Elramadi, Mohammed Radi, Hasan Abu Hejleh, Aiman Mudawi, Ahmed Mounir Elsayed

**Affiliations:** Hamad Medical Corporation, Doha, Qatar

**Keywords:** Osteochondroma, Regression, Exostosis, Benign bone tumor, Vanishing tumors

## Abstract

Osteochondromas are the most common benign tumors of the bone. Mainly these lesions affect the long-bone metaphysis and usually are asymptomatic. When complications develop from these lesions, then they become symptomatic and surgical resection may become indicated. Spontaneous resolution of osteochondroma is rare. There have been fewer case reports about this condition. We are reporting 16 years old, male, who sustained direct trauma to his shoulder and presented with fracture at the base of a solitary osteochondroma. Complete resolution of the lesion occurred without any surgical intervention 18 months following the fracture.

## Introduction

Osteochondromas are the most common bone benign tumors. It is defined as a cartilage-capped bony projection on the bone external surface, secondary to a perichondral ring defect (ring of Ranvier) and mainly affecting long bone metaphysis [1].

The vast majority of osteochondroma, are solitary with only around 15 % of them can be multiple in the context of hereditary osteochondromatosis. [2,3] usually these benign tumors are asymptomatic, and when it became symptomatic generally it arise from a complication such as fracture, mechanical joint problem or neurovascular entrapment and malignant transformation. [[2], [3], [4], [5]]

Treatment of osteochondromas consists of close observation with routine clinical and radiological follow up, whereas surgical resection is indicated for symptomatic cases. [[6], [7], [8], [9]] However Spontaneous osteochondroma regression is a rare occurrence, we are presenting a case of complete regression of a solitary osteochondroma at the humerus following fracture at its base in a 16 years old, boy.

## Case presentation

Sixteen years old, healthy, active boy, with no known co-morbidities, and no family history of any inherited disease. He attended our Emergency room with history of left mid-arm localized pain and swelling over a 2 weeks duration following a direct trauma. During this period the arm was tender to touch with increasing swelling around the arm. There were no constitutional symptoms, and he did not have any symptoms in the same arm prior to the trauma.

On examination the boy was conscious and oriented, with stable vital signs. Local examination of the left arm showed proximal arm diffused swelling with soft compartments, while on palpation he had tender, fixed, non-mobile, firm mass at the lateral aspect of the left proximal humerus just above the deltoid muscle insertion. The overlying skin was normal and there were no signs of any ongoing infection. However, the patient had full active and passive range of motion of the shoulder with no signs of any shoulder instability and intact distal neurovascular examination. No other sites were involved with similar mass or swelling.

**Fig. 1:**
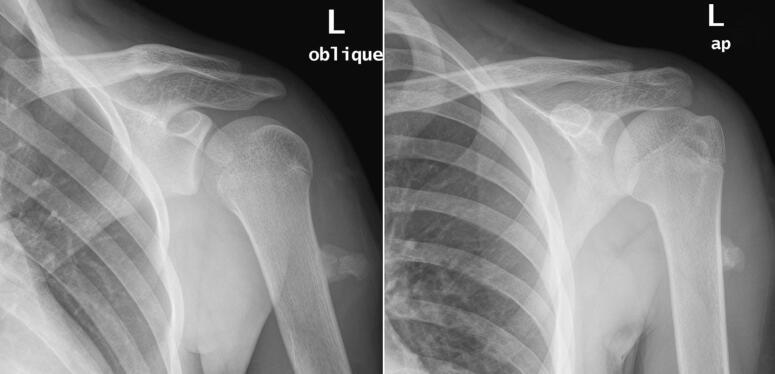
Anteropostreior (AP) & oblique views of the left humerus 2 weeks following a direct trauma, showing solitary pedunculated osteochondroma at the humerus proximal third with features of callus formation over a fracture stump.

Radiographic examination reviled solitary pedunculated osteochondroma at the proximal third of the humerus with features of callus formation over the fracture stump. (Fig. 1) The patient was given arm sling for comfort and discharged home with follow up appointment at the orthopedics oncology clinic. During the follow up the patient had improved symptoms with complete healing of the fracture at 4 months. The decision was made to manage the tumor conservatively and review the patient again in 1 year with repeat radiographs for consideration of surgical removal.

**Fig. 2:**
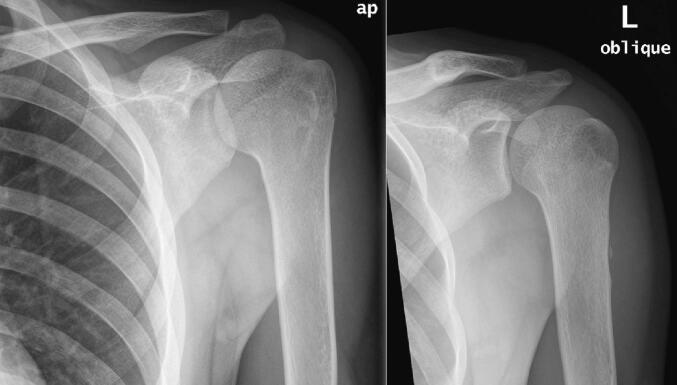
Anteropostreior (AP) & oblique views of the left humerus 12 months following the trauma, showing almost complete regression of the lesion with only a small boney remnant and scalloping of the posterolateral cortex at the site of the lesion.

**Fig. 3:**
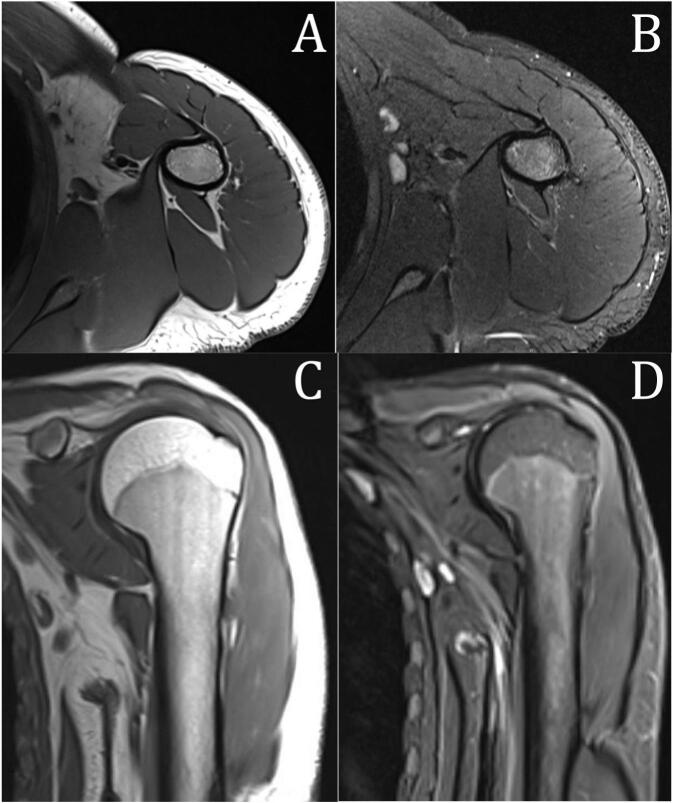
MRI of the left proximal humerus, A & B: T1 & T2 fat sat axial cuts showing almost complete resorption of the osteochondroma with only bony remnant at the posterolateral aspect of the proximal third of the humerus. C & D: T1 & T2 fat sat coronal cuts showing almost complete resorption of the lesion.

**Fig. 4:**
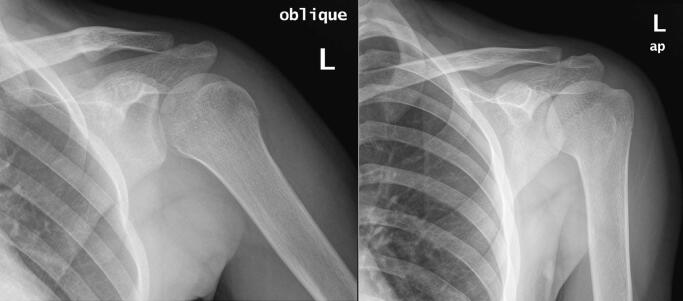
Anteropostreior (AP) & oblique views of the left humerus 18 months following the fracture, showing complete resolution of the osteochondroma and bone remodeling.

14 months following the injury he remained asymptomatic, and a new X-ray showed almost complete regression of the lesion with only a small bony remnant and scalloping of the posterolateral cortex at the site of the lesion. (Fig. 2) subsequent MRI of the humerus was obtained to confirm the findings. (Fig. 3) Finally 18 months following the fracture, the patient was symptoms free and a new X-ray showed complete resorption of the lesion with no bony remnant. (Fig. 4).

## Discussion

Osteochondromas are slow growing benign tumors that are usually asymptomatic. However, spontaneous regression of such tumor is a rare entity and there have been only few reported cases. Most of the reported cases were sessile and predominantly reported in young males [[9], [10], [11], [12], [13], [14], [15], [16], [17], [18], [19], [20], [21], [22], [23], [24], [25], [26], [27], [28]]. Radiographic evaluation was sufficient to establish the diagnosis in majority of the cases, and treatment was consistent of observation with routine clinical and radiological follow up [[9], [10], [11], [12], [13], [14], [15], [16], [17], [18], [19], [20], [21], [22], [23], [24], [25], [26], [27], [28]]. However, some authors recommended other modalities for confirming the diagnosis including CT scan and MRI. [17,21,24] Radiographic evaluation for our patient was sufficient for the diagnose, nevertheless an MRI was done during the follow up to confirm the resorption of the lesion.

The humerus was the most common sit of involvement followed by the distal femur and proximal tibia was the third most common site. [25] The exact cause for the spontaneous resolution of the lesion is not yet well known. Some theories have been proposed for the mechanism by which spontaneous regression of the osteochondroma occurs. Claikens & Paling et al., suggested that the growth of the lesion stops prior to the skeletal maturity, thereby leading to incorporation of the lesion into the adjacent growing bone. [14,23] Some authors have contributed that to the presence of a pseudoaneurysm accompanying the osteochondroma leading to the complete resolution of the tumor. [28]

However, it has been hypothesized that a combination of vascular compromise followed by bony repair secondary to fracture is the mechanism by which resorption occurs, as trauma can interrupt the growth of the lesion's cartilaginous cap and incorporating it as part of bone healing process, [13]. In the presented case this theory is mostly likely method of tumor regression, as there was clear history of trauma and the patient presented with evidence of with fracture line at the base of the lesion with callus formation on radiographs. Therefore, a period of observation in a skeletally immature patient with solitary osteochondroma is justified since some of these lesions initially mature and then regress. However, in symptomatic patients' surgical intervention maybe delayed until skeletal maturity and complete bone growth.

## Conclusion

Although rare, spontaneous regression of solitary osteochondromas in a skeletally immature patient may occur. It has been reported that an exostosis base fracture can lead to complete resorption of the tumor during fracture healing. Therefore, a period of conservative treatment should be considered in such cases. However, surgical resection should be reserved for the significantly symptomatic lesion.

## Statement of ethics

This article was Approved by the local Medical Research Center. Hamad Medical Corporation, Orthopedics Department, Doha, Qatar.

## Funding source

Open access funding was provided by the Qatar National Library.

## Consent

Written informed consent was obtained from the patient for publication of this case report and accompanying images.

## Declaration of competing interest

The authors have no competing interests to declare.
